# Growing couch potatoes? The impact of COVID-19 in the light of personal values in Hungary

**DOI:** 10.1186/s40100-022-00217-8

**Published:** 2022-03-31

**Authors:** Viktória Szente, Imre Fertő, Zsófia Benedek

**Affiliations:** 1 Hungarian University of Agriculture and Life Sciences, Kaposvár, 7400 Hungary; 2 Centre for Economic and Regional Studies, Budapest, 1097 Hungary; 3 John von Neumann University, Hungarian National Bank—Research Center, Kecskemét, 6000 Hungary

**Keywords:** Local food, Consumer segment, Portrait values questionnaire, Coronavirus, Lockdown, Hungary

## Abstract

The spread of the COVID-19 pandemic has unprecedentedly affected consumer behaviour. This paper reflects on changes in food consumption, buying, and training (working out) habits using a representative sample of 1000 Hungarian consumers and identifies consumer segments with unique needs based on personal sources of motivation. The widely known Schwartz Portrait Value Questionnaire was used to characterize individual value profiles. Employing k-medians clustering, three clusters were identified. “Business-as-usual People” managed to maintain their daily routines. The frequency of buying local food decreased the least among them. The sedentary lifestyle of the next cluster, “Inactive savers”, appears to have been accompanied by a lower level of food intake. Accordingly, this group was possibly less subject to the negative impacts of obesity, but more in need of psychological support to avoid devastating mental health outcomes. The third cluster initially appeared to encompass “Couch Potatoes” based on their COVID-induced lifestyles. However, the analysis of the value portraits of the latter showed that they were not couch potatoes at all, but rather active and proactive “Stay-at-home Businesspeople” who may benefit from guidance regarding how to manage the increase in housekeeping and childcare, and how to become more physically active in the home environment. The results are valuable from both a scientific and practical perspectives.

## Introduction

The pandemic and health crisis related to the Coronavirus disease of 2019 (COVID-19) has profoundly changed economic life, business, and consumer habits. With the initiation of the COVID-related lockdown, consumers had to redefine and adapt their daily routines and lifestyles as they stayed at home for a long period. Habits related to eating, shopping, dressing, and working out changed, among other factors, while the increase in importance of the role of home entertainment and digital experiences affected social life (Laguna et al. 2020; Leone et al. 2020; Naja and Hamadeh 2020). During the first weeks of the pandemic, grocery retailers encountered overwhelming demand paired with panic buying, resulting in empty shelves (Hall et al. 2020a, b). To reduce the risk of exposure, many consumers shifted to online shopping instead of entering retail stores (Chang and Meyerhoefer 2020), while many governments introduced dedicated shopping times for elderly people. Restaurants mainly offered home delivery services or take-away options (Poelman et al. 2020). Gyms and other sports facilities also closed their doors to decrease human contact (Chtourou et al. 2020; Huszka et al., 2020). Citizens in almost every country in the world were allowed to do some individual sports outside or to take family walks (Poelman et al. 2020), but otherwise home workouts remained the only alternative for physical activity. Institutes of education suspended in-person classes and switched to online courses (Ben Hassen et al. 2020; Viner et al. 2020). Many people increased the time they spent on digital activities such as learning, being entertained, working, and socializing (GWI 2020; Sharma et al. 2020). These behaviours often led to sedentarism—too much screen time, and way too much sitting down (Grenita Hall et al. 2020a, b; Romero-Blanco et al. 2020; Zheng et al. 2020). This increase in inactivity may well contribute significantly to the incidence of cardiovascular disease, osteoporosis, and adult-onset diabetes, among other health issues (Barone et al. 2020; Mattioli and Ballerini Puviani, 2020; Peçanha et al. 2020; Zhang and Chen 2020). The lockdown has also been associated with more direct lifestyle-related behavioural changes, including self-reported weight-gain due to snacking, elevated stress, and less time sleeping (Sidor and Rzymski 2020; Xiang et al. 2020), as well as an increase in the consumption of unhealthy food, uncontrolled eating, snacking between meals, and the consumption of a greater number of main meals (Carroll et al. 2020; Di Renzo et al. 2020).

COVID has disrupted global food systems, too, especially food production and logistics chains (Ben Hassen et al. 2020; Elleby et al. 2020; OECD 2020). Short food supply chains often proved to be an important means of ensuring food security (Cappelli and Cini 2020; Henry 2020; Leone et al. 2020), although heterogeneous outcomes have been experienced in this regard. For example, results from a study of Qatari consumers revealed that 34% of respondents purchased local food more often. Motivations included safety considerations, as consumers increasingly wanted to know where the food they buy came from (Ben Hassen et al. 2020). On the other hand, a study from Vermont reported that many people, mostly food-insecure consumers, decreased their purchase of fresh local foods products due to their limited access to outlets after the closure of farmers’ markets (Niles et al. 2020).

The aim of this piece of work is to broaden the literature with an analysis of the impact of the COVID-related lockdown on consumer behaviour, with special emphasis on attitudes towards local food and other food purchases and physical activity. Our interest was in identifying the underlying psychological profiles of different consumer segments to better understand the drivers of behavioural attributes. The empirical analysis focuses on Hungarian consumers. Here has to be mentioned that the mortality rate in Hungary was among the lowest in international comparison (Cao et al. 2020), while related restrictions were gradually lifted from 4 May 2019, after the first wave. COVID has had severe impacts on various consumer habits. As the interactions between consumer choices, lifestyles, and values are the analytical interest of this paper, some considerations about human values and their measurement are briefly introduced in the following section.

### Theoretical background: the Schwartz theory of basic human values

Values play an important role in sociology, psychology, and anthropology, while their use in analysis is increasingly acknowledged in the field of economics, too. Values are used to characterize cultural groups, societies, and individuals, to trace changes over time, and to explain the motivational bases of attitudes and behaviour (Schwartz 2012). Values are broad, motivational constructs that express what is important to humans. Depending on values, products, objects, people, services, and events are evaluated differently. The values of people are central to their identity and concept of self (Rokeach 1973; Schwartz 2012). Furthermore, values reflect socially desirable concepts used to represent motivations and goals.

The Schwartz Theory of Basic Human Values (TBHV, Schwartz 1992) is one of the most influential constructs of the research of values (Verkasalo et al 2009) and is used in a number of contexts, from religious studies (Saroglou et al. 2004) to trust in institutions (Devos et al. 2002) and the prediction of behaviour (Bardi and Schwartz 2003). The Theory of Basic Human Values has been established in behavioural research as a comprehensive model that is stable across cultures and which can be used to predict a series of external constructs (Saroglou et al. 2004).

According to the TBHV, values are “individual concepts about a trans-situational goal that express an interest included in a motivational (goal) domain valued by the range of importance and that act as a guiding principle in the life of persons” (Schwartz 1992, cited in Giménez and Tamajón, 2019). Based on the type of goal, or the underlying motivation, ten broad values can be distinguished (A1 in the Appendix). The theory also accounts for the dynamic relations (conflicts and congruity) among the values (Schwartz 2012).

**Fig. 1:**
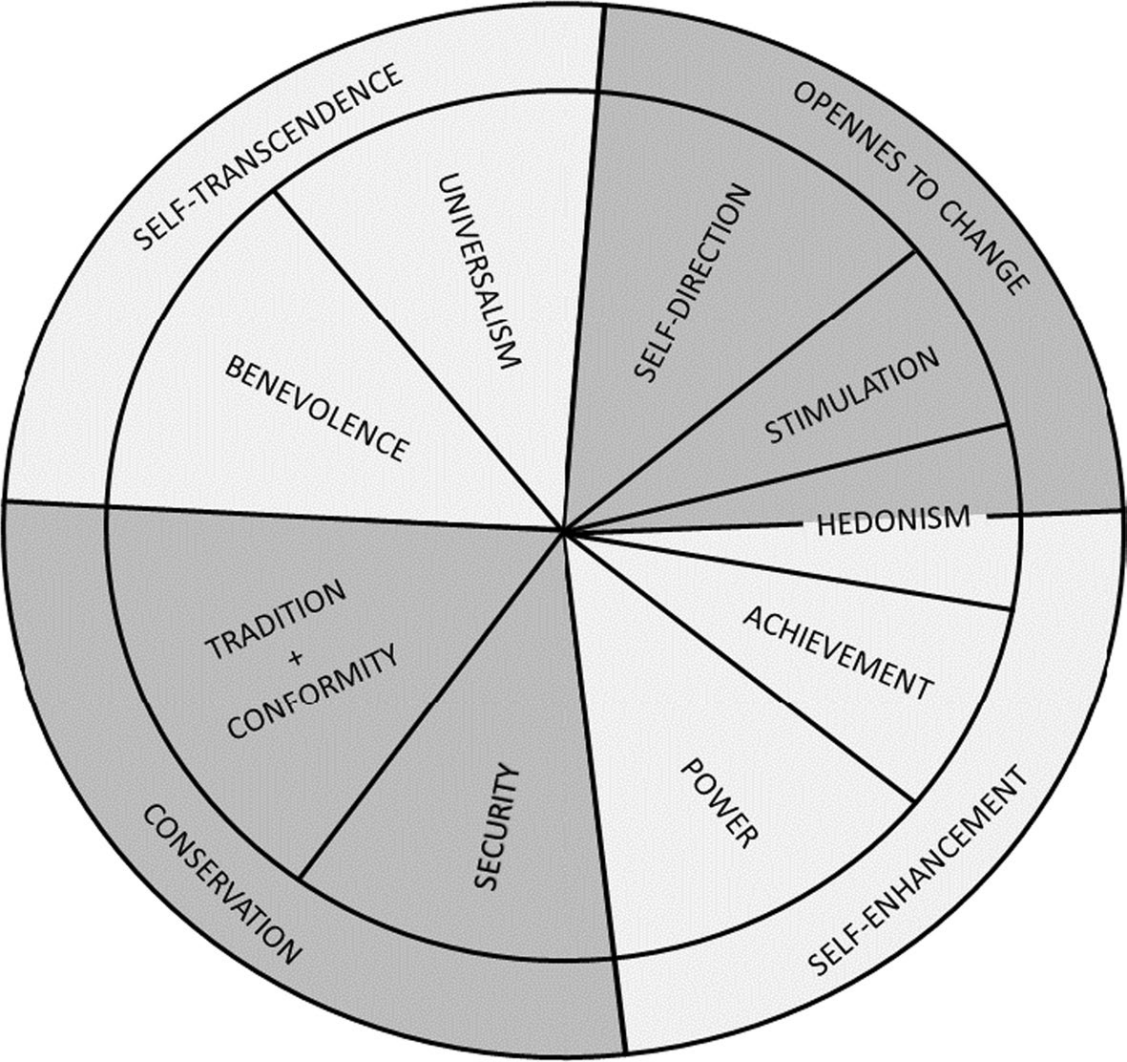
The Schwartz theory of basic human values. *Source*: Schwartz (1992), cited in Giménez &Tamajón, 2019, Fig. 2

Figure 1 shows the ten values differentiated into pairs of opposite main dimensions.

The four main dimensions are arranged in a circular model (Hinz et al. 2005). Adjacent value types are thought to be the most compatible with one another, while the greatest conflicts are between opposing values. The opposition of Openness to Change to Conservation values represents the confrontation between values that are based on personal independence and openness to change, and values that emphasize order, self-restriction, and resistance to change. Hedonism shares elements of both openness to change and self-enhancement, while tradition and conformity are located on the same wedge as they share the same broad motivational goal (subordination of the self in order to ensure stability). The other dimension reflects the opposition between the values of Self-transcendence (concern for the others) and Self-enhancement (which emphasizes the importance of individual success and dominance).

There are two major methods for measuring basic values: the Schwartz Value Survey (SVS) and the Portrait Values Questionnaire (Schwartz et al. 2001). The latter was found to be easier to implement and its outcomes proved to be more consistent (Lusk et al. 2006), thus it was used in the analysis described in this paper.

While the original version of PVQ comprised 29 items for measuring individual human values (Schwartz et al. 2001), a new, simplified, and less abstract 21-item PVQ was developed for the purpose of the European Social Survey (Verkasalo et al. 2009). Each item consists of a description (a “portrait”) of a person. All the values are represented by two sentences, except for “universalism”, which is measured using three items. Respondents are asked to assess the similarity of the portrayed person to themselves on a six-point scale.

The findings reveal substantial differences in the value priorities of individuals; however, the average value priorities of most societal groups exhibit a similar hierarchical order (Saroglou et al. 2004; Verkasalo et al. 2009).

The following section describes the importance of values from the perspective of consumer behaviour research.

### Values, lifestyle, and segmentation

The choice of consumers to use specific products and services clearly reflects their lifestyle (Levy 1999). Specific values have been linked different purchasing and consumption decisions (Kahle et al. 1986; McCarty and Shrum 1993). Knowledge of values and their relationship to individual consumption targets can help predict purchasing behaviour and the use of products (Herrero et al. 2014).

Several studies have confirmed the relationship between values and lifestyles, the selection of food products (Askegaard and Brunsø, 1999; Brunsø et al. 2004; Renko et al. 2014), food-related behaviour (Nijmeijer et al. 2004; Szakály et al. 2012), and physical training (Imas et al. 2018; Kangasniemi et al. 2015). Sarmugam and Worsley (2015) identified consumer segments based on impulsivity and level of involvement with food and characterized the dietary behaviour of each segment, while Valentine and Powers (2013) associated lifestyle segments in Generation Y with specific forms of media use.

Food consumption behaviour and lifestyles apparently changed after the outbreak of COVID (Ben Hassen et al. 2020; Laguna et al. 2020; Poelman et al. 2020; Sánchez-Sánchez et al. 2020; Romeo-Arroyo et al. 2020). An increasing number of studies have concluded that different consumer segments might need specific guidance about how to avoid the undesirable health impacts of lockdown (Al-Musharaf 2020; Romero-Blanco et al. 2020; Vancini et al. 2020). Although the role of psychological factors in lifestyle changes (e.g. the impact of stress on dietary choices and junk food consumption) is sometimes emphasized (see e.g. Di Renzo et al. 2020), systematic discussion of the psychological needs of individuals who have specific types of lifestyles is lacking. However, such needs should be taken into account when strategies for promoting well-being are formulated (Antunes et al. 2020).

## Materials and methods

### The sample and key variables

In order to analyse changes in food consumption and training habits following the outbreak of COVID-19 in Hungary, a questionnaire was designed. The questionnaire was approved by the Ethical Committee of the Centre for Economic and Regional Studies, Hungary, and informed consent was obtained from all the respondents. Primary data were collected through the phone in May 2020 by a professional survey company. The survey is representative of the Hungarian population in terms of demographic characteristics (gender, age, type of settlement, education) and regional distribution according to the data of microcenzus in 2016. To this end, 104 settlements were first identified, and then 87,400 results were selected based on the surnames who could be visited there. This was followed by multi-stage quoting, first based on regional distribution/settlement type and 4-dimensional matrix (gender/age/school/settlement type). Only Hungarians above 18 years were involved in the study. The sample size is 1000.

Next, the key variables are described briefly. Data were collected about the socio-demographic and economic characteristics of the respondents and their households. Another section was used to collect values that replicated the Portrait Value Questionnaire (PVQ) of Schwartz (1992). The list of questions published by Pascucci et al. (2016) was translated and used (Table 9 in the Appendix). The 21 answers were used to create the Schwartz portrait of respondents consisting of ten values by calculating the mean scores collected from pairs of questions (or a triplet of questions for one value). The following section of the survey was dedicated to the identification of the habits and lifestyle of respondents before and after the outbreak of the pandemic. Respondents were asked to express how often they had done and were doing specific things on a Likert scale (1: daily; 2: many times a week; 3: once a week; 4: many times a month; 5: once a month; 6: occasionally; 7: never). Changes in habits and lifestyle were defined as the differences in the means compared using paired t tests. Only variables that were significant at least at the 1% level were involved in the statistical analysis. With respect to lifestyles, lunch is typically considered the main (warm) meal in Hungary, even on weekdays, although many families enjoy a light warm dinner.

### Statistical analyses

Cluster analysis with k-medians was employed to identify consumer groups with respect to lifestyle elements—specifically, food consumption and training habits. The Calinski–Harabasz pseudo-F index (Caliński and Harabasz 1974) was used to determine the number of clusters. Classifications of two to four clusters were considered; the interpretation of more clusters might have been challenging. Nonparametric Kruskal–Wallis tests were used for cross-validation when the means in the different clusters were tested in relation to the variables used to describe demographic variables and variables of the Schwartz portrait profile; these variables were not involved in the clustering itself.

Finally, we investigated the impacts of personal values on the cluster grouping. We also checked whether our data meet with the independence of irrelevant alternative (IIA) assumption for the multinomial logit model. Logit analysis has been widely used in social sciences to determine whether there are any significant relationships between two or more variables using scales, as well as to contrast the differences between any two levels of an independent variable (Birda et al., 2010). We computed three tests of the independence of irrelevance alternatives: Hausman-McFadden the Small-Hsiao test, and the SUEST version of the Hausman-McFadden test. All tests suggest that no evidence for the IIA assumption has been violated. The hypothesis that all the coefficients are simultaneously equal to zero is rejected by the LRχ2-test for both outcomes (inactive savers = 2 and coach potatoes = 3).

### Characteristics of the sample

Table 1 displays the descriptive statistics for the demographic and economic variables.

**Table 1:** Demographic variables: descriptive statistics

Variables	Obs	Mean	SD	Min	Max
Gender (1: female, 2: men)	1000	0.55	0.50	0	1
Age (years)	1000	51.92	16.63	18	95
Education^1^	1000	2.73	0.94	1	4
Dwelling size (m^2^)	931	88.50	34.86	20	320
Per capita income (EUR)^2^	1000	254.9	402.1	0	6147.2

^1^The level of education was expressed on a scale of four: 1: completed primary education or lower; 2: completed vocational education; 3: completed secondary education; 4: completed higher education

^2^Average after-tax income in Hungary was 244,600 HUF (752 EUR) in 2019 (data from the Hungarian Central Statistical Office)

## Results and discussion

First we introduce the main data and basic statistical connections. Table 2 shows the descriptive statistics of the Schwartz portrait values.

**Table 2:** Schwartz portraits variables: descriptive statistics

Value group	Value	Obs	Mean	SD	Min	Max
Self-enhancement	Power	992	3.47	1.16	1	6
	Achievement	994	2.78	1.22	1	6
	Hedonism	993	2.11	0.86	1	6
Self-transcendence	Universalism	982	1.66	0.69	1	6
	Benevolence	994	1.75	0.76	1	6
Openness to change	Stimulation	996	3.00	1.17	1	6
	Self-direction	991	1.99	0.87	1	6
Conservation	Tradition	993	2.24	1.00	1	6
	Conformity	994	1.95	0.94	1	6
	Security	976	1.68	0.82	1	5

In order to assess the outcomes in the light of other studies, our results were compared to those of Pascucci et al. (2016), who studied personal values in the context of food to reveal a portrait of consumers in general and of consumers participating in alternative food networks in Sicily, Southern Italy, using a non-representative but large sample (*N* = 303). The Italian data were collected in 2012, and thus the related condition concerning comparability (data contained in studies intended to be compared should not be collected more than approximately a decade apart–see Saroglou et al. 2004) was not violated. The scores of the Hungarian consumers lie mostly in between the scores for the two Italian groups. Differences appeared with respect to Hedonism (3.03–2.80 for Italian consumers), Tradition (2.67–2.32 for Italian consumers), Conformity (2.88–2.70 for Italian consumers), and Security (2.49–2.22 for Italian consumers). As hedonism is traditionally found to be an important element of the Mediterranean identity (see e.g. Petruzzellis et al. 2016; Petruzzellis et al. 2017), the relatively low Hungarian Hedonism score (higher attribution) is surprising and is possibly a psychological impact of the pandemic involving an increase in focus on the well-being of the self during isolation. Low Conservation scores are in line with expectations: Verkasalo et al. (2009) found that the increased importance of Conservation values in relation to the opposing Openness to Change values was typical of countries with a former communist regime. Additionally, subordination, which is the core motivation behind the values of Tradition and Conformity (Schwartz 2012), as well as the discipline of citizens, was potentially important conditions for successfully combating the virus situation during the first wave (Cao and Liu 2020). The very high importance awarded to Security might also be a consequence of the then-ongoing first wave.

Though the Portrait Value Questionnaire was originally created to be general and context-free, thus permitting cross-cultural comparisons (Schwartz et al. 2001; Verkasalo et al. 2009), it probably gives the best results if only the structures of personal value systems are evaluated (de Wet et al. 2019), while the differences in means might reflect differences in specific life contexts. Therefore, even if the broad contexts of the two studies (“food”) match, the interpretation we provide should be regarded with caution, especially as the Hungarian data were collected during a crisis. However, the higher attribution of Conservation and Self-enhancement values of Hungarian consumers in the midst of the COVID pandemic compared to those of the average Italian consumer, and the lower attribution of the opposing Openness to Change and Self-transcendence values, may be considered feasible. The relative position of Conservation has already been discussed, but further research is required to reveal whether the other differences are general, or consequences of the pandemic.

Table 3 shows the lifestyle-related variables and their descriptive statistics.

**Table 3:** Variables used for the COVID-related lifestyle segmentation of Hungarian consumers

Variable	Min	Max	Obs	Mean^1^	SD	Obs	Mean^1^	SD	*t* test (p value)
			Pre-COVID scores			Post-COVID scores			
Buying local food	1	7	993	2.96	1.3	993	3.29	1.53	0.0100
Cooking	1	7	995	2.08	1.49	995	1.98	1.37	0.0000
Eating convenience bakery products for breakfast	1	7	998	3.84	2.01	993	4.49	2.07	0.0000
Ordering lunch	1	7	998	5.91	1.61	999	6.06	1.65	0.0000
Ordering dinner	1	7	998	6.3	1.26	999	6.39	1.26	0.0036
Consuming soft drinks	1	7	998	4.19	2.32	998	4.32	2.38	0.0008
Enjoying light training	1	7	998	2.25	1.59	998	2.46	1.83	0.0003
Doing an intense workout	1	7	997	4.68	2.41	997	4.84	2.42	0.0076

^1^Lower values indicate higher frequency

In most households, the individuals responsible for cooking (mostly women; EuroStat 2019) cook regularly, many times a week. Enjoying light training (such as having a walk, gardening, and going on outings) is also a frequent activity among Hungarians. However, doing intensive workouts regularly is much less typical; not only the mean score but also the standard deviation is also relatively high, indicating a very uneven distribution for this parameter. This is in line with expectations: according to Rurik et al. (2014), obesity is considered a public health threat in Hungary. Similarly to the findings of Fricz Szegedyné et al. (2020), local food appears to be quite popular; households buy local food products once a week on average. Though access to local food is generally considered a component of obesity control (see e.g. Morland and Evenson 2009; Spence et al. 2009), a systematic review by Cobb et al. (2015) found limited evidence for this association. Buying food for lunch or dinner that was prepared by someone else is very rare among Hungarians in general, possibly due to financial considerations; similarly, eating out is not a form of socialization that is as common as among, for example, Spanish consumers (Romeo-Arroyo et al. 2020). Regarding changes in food consumption and training habits after the outbreak of COVID, only the frequency of cooking increased as people spent more time at home following the restrictions on movement and increased use of the home office. With more frequent cooking, the originally low frequency of buying prepared food (ordering of lunch and dinner from restaurants or canteens) decreased further. The most remarkable decline was in the eating of convenience food for breakfast. Originally, the distribution of the frequency of this variable was quite uneven: 57% of the sample ate convenience bakery products for breakfast at least once a week before the pandemic, while many never enjoyed a convenience breakfast. With many people staying at home, the frequency obviously declined.

The second biggest drop concerned buying local food. Due to the increase in isolation, the closure of many markets (or their limited availability following the introduction of the elderly-only time window), consumers had fewer opportunities to purchase food that involved direct contact with producers. Though data were not collected about the size of purchases, it is theoretically possible that more items were purchased per occasion. However, Benedek et al. (2020) revealed that roughly 60% of Hungarian small-scale producers experienced economic losses, and thus overall sales volumes across Hungary appear to have declined, although interest in some specific short food supply chains (such as consumer purchase groups and box schemes) increased sharply (Nemes et al. 2020). According to Authors (under review), the boom in these flourishing short food supply chains involved only a limited number of producers who mostly operated at specific, favorable locations (such as in the vicinity of big cities).

Identifying changes in these different habits was the core reason for the following clustering exercise. According to the Calinski–Harabasz pseudo-*F* index, a definition of three clusters was the optimal solution. Table 4 shows the lifestyle profiles of the clusters.

**Table 4:** Change in activity by cluster (N = 972)

Activity	Business-as-usual people	Inactive savers	Couch potatoes	Kruskal–Wallis (*p* value)
Buying local food	− 0.19	− 0.65	− 0.50	0.0001
Cooking	0.03	0.01	0.52	0.0264
Eating convenience bakery products for breakfast	0.03	− 3.53	0.34	0.0001
Ordering lunch	− 0.07	− 0.63	0.14	0.0001
Ordering dinner	− 0.04	− 0.31	− 0.04	0.0123
Consuming soft drinks	− 0.05	− 0.27	− 0.30	0.0533
Enjoying light training	0.10	− 0.02	− 2.31	0.0001
Doing an intense workout	0.22	− 0.29	− 2.12	0.0001
N	664	197	111	–

The most populous cluster defines what we call “Business-as-usual (BAU) people” (68.3%). These individuals maintained most of their routines at a similar frequency as before the outbreak of COVID. The frequency of buying local food decreased the least among them. Furthermore, there is not a big difference in the habit of ordering meals, implying that they can be a steady target group for restaurants. Moreover, it was this group (and only this one) that increased the frequency of training activities, be this light exercise or (especially) intense workouts.

People in the next most populated cluster (20.3%), called “Inactive savers”, reduced all the activities that include spending covered by the survey. They bought local food less frequently and bought prepared food (and soft drinks) less often. The frequency of doing intense workouts also declined for this group. The reduction of activity related to food purchases was not accompanied by a significant increase in the frequency of cooking. As practically none of the activities increased in frequency, it is challenging to evaluate the lifestyles and interests of members of this group during COVID, and and hence they were described as inactive people.

Members of the third and smallest cluster (11.4%), “Couch potatoes”, decreased their rate of physical exercise significantly. Unlike “inactive savers”, they cooked more often, and usually ate more convenience bakery products and ordered from restaurants. Accordingly, and also due to the decline in working out, this group is most likely to encounter health problems and an increase in body weight (as in Sánchez-Sánchez et al. 2020; Vancini et al. 2020).

While the socio-economic and value-based variables were not involved in the clustering exercise, they were used to validate the results of the clustering, permitting further differentiation between clusters. Table 5 displays cluster differences in socio-economic terms.

**Table 5:** Socio-economic profiles by cluster (only significant differences are shown)

Variable	BAU people	Inactive savers	Couch potatoes	Kruskal–Wallis (*p* value)
Education	2.66	2.94	2.94	0.0005
Dwelling size (m2)	88.37	92.40	84.64	0.0777
Per capita income (HUF)	80,265	99,437	103,367	0.0482

“BAU people” appeared to have the lowest level of education, and the lowest per capita income. The variables were associated with the highest values in the case of the “Couch potatoes”, who tended to live in the smallest dwellings. “Inactive savers”, who were also characterized by a higher level of education, lived in the biggest dwellings. Table 6 shows the value-related differences that were significant.

**Table 6:** Schwartz portraits profiles by cluster (only significant differences are shown)

Value group	Value	BAU people	Inactive savers	Couch potatoes	Kruskal–Wallis (*p* value)	Definition of value construct^1^
Self-enhancement	Power	3.49	3.63	3.02	0.0002	“Social status and prestige, control or dominance over people and resources.”
	Achievement	2.81	2.82	2.38	0.0025	“Personal success through demonstrating competence according to social standards.”
	Hedonism	2.11	2.26	1.91	0.0052	“Pleasure and sensuous gratification for oneself.”
Openness to change	Stimulation	3.01	3.18	2.60	0.0001	“Excitement, novelty, and challenge in life.”

^1^Definitions taken from Schwartz et al. (2012), Table 1, p. 521

^2^lower values indicate higher acceptance

The most remarkable differences appeared in the Self-enhancement value construct and, partly, in the Openness to Change value group, and always in relation to Inactive Savers (highest scores, lowest attributions) and “Couch potatoes” (lowest scores, highest attributions). The profile of “Couch potatoes” may be described as follows. In comparison to the sample average (depicted in Table 1) their scores indicate that they are active—indeed, proactive—people who are used to taking control of their lives. Unlike “BAU people”, they did not maintain pre-COVID routines as much as possible, but adapted to and took advantage of the situation while achieving gratification. Thus, they can be characterized as positive thinkers who look for and accept challenges. Such a profile is very different to the idea of a “real”, lazy couch potato, despite the apparent lifestyle characteristics, thus an alternative name, “Stay-at-home Businesspeople”, is suggested to account for their higher level of education and income. These individuals are more likely to be white-collar workers who in the specific COVID-related situation were able to work from home, but the increase in isolation created more "home-care" (and child-care) tasks for them, thus they had no time (or proper equipment, space, etc.) to do physical exercise, although they possibly desired a healthier lifestyle. Thus, a new market gap seems to have emerged for efficient time management instruments (from organizers to quick-to-prepare recipes), and guidance regarding how to reengage with physical exercise.

The other extreme is that of “Inactive savers”. Also highly educated people, these individuals place less importance on status, power, achievement, and challenges. The former characteristics are contradictory; these individuals may have been negatively psychologically influenced by COVID-related conditions. They also seem to have stayed at home, based on the decrease in the frequency of activities related to eating and working out. This group seems to be most in need of psychological support to maintain their emotional and mental health, while their sedentary lifestyles appear to be accompanied by a decrease in food intake, thus this group will possibly be less prone to the negative impacts of obesity.

The lower level of education of “BAU people” indicates that they are more likely to be engaged in manual work that cannot be done from home. As they continued to go leave home, the maintenance of their daily routines is not surprising. In terms of values, they are more similar to Inactive Savers, thus they are not considered to be enterprising.

The contradictions revealed by the clash of observed lifestyle characteristics and value profiles indicate that—especially in such a crisis situation—analysis of only one aspect of life can be easily misleading. Although enhancing lifestyle-related messages might be an important means of helping individuals cope with difficulties—as proposed by e.g. Al-Musharaf (2020), Romero-Blanco et al. (2020), Vancini et al. (2020) and others, in accordance with Antunes et al. (2020), we suggest that this might not be enough to achieve desired goals. Accordingly, psychological dimensions and other factors (such as age, gender, and family status) should also be taken into account when messages are targeted, especially concerning mental health.

The impact of values on cluster grouping was assessed through a multinomial logit model. Concerning the outcome variable, the reference group is represented by the BAU people. The estimation reinforces the results of cluster analysis. Regarding Schwartz values, the coefficients of Power, Achievement, Hedonism, and Stimulations are significant (Table 7).

**Table 7:** Results of the multinomial logit model

Schwartz values	Inactive savers	Couch potatoes
Power	0.194*	0.276**
	(0.117)	(0.135)
Achievement	0.206*	0.096
	(0.124)	(0.140)
Hedonism	0.161	0.313*
	(0.170)	(0.190)
Stimulation	0.204*	0.308**
	(0.120)	(0.137)
Self-direction	0.122	0.124
	(0.165)	(0.185)
Universalism	− 0.259	− 0.241
	(0.200)	(0.234)
Benevolence	− 0.273	− 0.231
	(0.178)	(0.205)
Tradition	− 0.006	0.014
	(0.125)	(0.146)
Conformity	0.017	0.032
	(0.149)	(0.171)
Security	− 0.136	− 0.240
	(0.151)	(0.177)
Constant	0.643	− 1.240**
	(0.468)	(0.559)
N	914	
Pseudo R^2^	0.029	

Standard errors are in parentheses, **p < 0.05, *p < 0.1

The probability of being an inactive saver or couch potato increases significantly with valuing power and stimulation. Achievement is positively associated only with inactive savers, while hedonism is with couch potatoes.

## Conclusions and suggestions

During a prolonged crisis, such as that created by the coronavirus pandemic, maintaining community health is of the highest importance. Besides reducing the infection rate, the physical and mental health of individuals should be taken into account to avoid undesired impacts in the medium or long term. The aim of this paper was to analyse the impact of COVID-19 on the lifestyle-related choices of a representative sample of Hungarian consumers, also incorporating personal values. The widely used 21-element Schwartz Portrait Value Questionnaire (PVQ) that creates psychological portraits of individuals according to 10 value groups was used to classify personal sources of motivation.

Our estimations identify three clusters. The most populous cluster, “Business-as-usual people”, describes those who could not stay at home, possibly due to the nature of their work (e.g. manual work). These individuals typically maintained their daily routines. The other two clusters were characterized by higher levels of education, but their coping strategies differed in terms of changes in lifestyles and values, while their sources of motivation were also dissimilar. “Inactive savers” were apparently stuck at home; the frequency of local food purchases as well as eating out and working out declined among them, while the PVQ revealed a decrease in proactivity that is surprising for people of their social status. This segment might be in need of psychological support to cope with the stressful situation. Analysis of the value portrait of the third cluster, in which individuals appeared to be “Couch Potatoes” at first sight based on their COVID-induced lifestyles, indicated that they are not couch potatoes at all, but on the contrary, active and proactive “Stay-at-home Businesspeople” who might benefit from guidance about how to manage the increase in their housekeeping and childcare load, and how to dial up their level of physical exercise to their previous level in a home environment.

To summarize, the clusters we identified would benefit from entirely different approaches concerning disease prevention. In terms of desirable community health outcomes, psychological and other factors should be taken into account, besides purely lifestyle nudges.

The strengths of this piece of work include its representativeness, and the application of the PVQ approach that makes the results comparable and generalizable. On the other hand, further research is needed to reveal the extent to which comparability is reduced by potentially altered perceptions and values in a crisis situation. Another limitation is that the frequencies of specific activities are self-reported, and our survey questions are clearly not appropriate for evaluating the physical (or mental) health of respondents. Nonetheless, our work is among the first studies to attempt an analysis of the personal values of individuals in this unprecedented situation.

The results are valuable from academic and practical perspectives, too. The change in personal values as a result of an unexpected lockdown situation has been documented. The different demands in the consumer segments have been highlighted, which might help manufacturers, traders and the stakeholders of the restaurants, canteens in planning their promotions, as well as the design of government precaution campaigns.

## Data Availability

The data sets used and/or analysed during the current study are available from the corresponding author on reasonable request.

## Appendix

Tables 8 and 9.

**Table 8:** Motivations (goals) of humans behind their values, and dimensions thereof. *Source*: adaptation based on Schwartz and Boehnke 2004; Giménez and Tamajón 2019

No	Schwartz Values	Motivation	Dimensions	Motivation
1	Self-direction	Independent thought and action-centered, creativity, exploration (creativity, freedom, independence, choosing one’s own goals, curiosity)	Openness to change	Controlling one's own impulses and behaviour, according to social norms and expectations
2	Stimulation	Excitement, novelty and challenge in life (daringness, variety and excitement in life)		
3	Hedonism	Pleasure or sensuous gratification for oneself (pleasure, enjoying life, self-indulgence)	Self-enhancement	Promoting self-interest at the expense of others, Emphasis on personal success and dominance over others
4	Achievement	Personal success through demonstrating competence according to social standards (ambition, success, capability, influence)		
5	Power	Social status and prestige, control or dominance over people and resources (authority, social power, wealth, preserving public image)		
6	Security	Safety, harmony, and stability of society, of relationships, and of self (family security, national security, social order, cleanliness, reciprocation of favors)	Conservation	Maintaining stability and security in relations with one's surroundings, with the emphasis on subservient self-repression, preservation of traditional practices, and emphasis on stability
7	Conformity	Restraining from action, inclinations, and impulses likely to upset or harm others and violate social expectations or norms (self-discipline, politeness, honoring parents and elders, obedience)		
8	Tradition	Respect, commitment, and acceptance of the customs and ideas that traditional culture or religion provide (devotion, respect for tradition, humbleness, moderation)		
9	Benevolence	Preservation and enhancement of the welfare of people with whom one is in frequent personal contact (helpfulness, honesty, forgiveness, loyalty, responsibility)	Self-transcendence	Promoting the wellbeing of society and nature above one's own interests, highlighting the acceptance of others as equals, as well as a concern for their wellbeing
10	Universalism	Understanding, appreciation, tolerance, and protection for the welfare of all people and for nature (equality, social justice, wisdom, broadminded, protecting the environment, unity with nature, a world of beauty)

**Table 9:** The Schwartz portrait values questionnaire and descriptive statistics

Description of people^1^	Obs	Mean	SD	Min^2^	Max
Thinking up new ideas and being creative is important to him/her. He/she likes to do things in his/her own original way	994	2.21	1.27	1	6
It is important to him/her to be rich. He/she wants to have a lot of money and expensive things	1000	4.04	1.44	1	6
He/she thinks it is important that every person in the world should be treated equally. He/she believes everyone should have equal opportunities in life	987	1.74	1.13	1	6
It is important to him show his/her abilities. He/she wants people to admire what he/she does	998	2.70	1.44	1	6
It is important to him/her to live in secure surroundings. He avoids anything that might endanger his/her safety	999	1.61	0.93	1	6
He/she likes surprises and is always looking for new things to do. He/she thinks it is important to do lots of different things in life	999	2.34	1.29	1	6
He/she believes that people should do what they’re told. He/she thinks people should follow rules at all times. even when no one is watching	997	1.84	1.07	1	6
It is important to him/her to listen to people who are different from him/her. Even when he/she disagrees with them. he/she still wants to understand them	997	1.94	1.07	1	6
It is important to him/her to be humble and modest. He/she tries not to draw attention to him/herself	997	2.28	1.21	1	6
Having a good time is important to him/her. He/she likes to “spoil” him/herself	997	2.40	1.20	1	6
It is important to him/her to make his/her own decision about what he/she does. He/she likes to be free and not depend on others	997	1.78	0.97	1	6
It is very important to him/her to help the people around him/her. He/she wants to care for their well-being	997	1.83	0.96	1	6
Being very successful is important to him/her. He/she hopes people will recognise his/her achievements	995	2.86	1.35	1	6
It is important to him/her that the government ensures his/her safety against all threats. He/she wants the state to be strong so it can defend its citizens	977	1.74	1.09	1	6
He/she looks for adventures and likes to take risks. He/she wants to have an exciting life	996	3.66	1.56	1	6
It is important to him/her always to behave properly. He/she wants to avoid doing anything people would say is wrong	995	2.06	1.18	1	6
It is important to him/her to get respect from others. He/she wants people to do what he/she says	992	2.91	1.45	1	6
It is important to him/her to be loyal to his/her friends. He/she wants to devote him/herself to people close to him/her	995	1.56	0.87	1	6
He/she strongly believes that people should care for nature. Looking after the environment is important to him/her	995	1.41	0.73	1	6
Tradition is important to him/her. He/she tries to follow the customs handed down by his religion or his/her family	994	2.20	1.30	1	6
He/she seeks every chance he/she can to have fun. It is important to him/her to do things that give him/her pleasure	994	1.82	0.91	1	6

^1^*Source*: Pascucci et al. 2016, p. 12

^2^The interpretation of the Likert scale: 1: Very much like me, 2: Like me, 3: Some-what like me, 4: A Little like me, 5: Not like me, 6: Not like me at all
